# Supplementary material for: Fungal mycelia and bacterial thiamine establish a mutualistic growth mechanism
Source: Life Sci Alliance. 2020 Sep 21;3(12):e202000878. doi: 10.26508/lsa.202000878 (PMC7574024; doi:10.26508/lsa.202000878)
Supplement: Supplementary file 11 [file LSA-2020-00878_TableS1.docx]

Table S1. *B. subtilis* genes differentially expressed in the co-culture condition*^a^*

| Gene name | Product | Fold change in co-culture |
| --- | --- | --- |
| Up regulated |  |  |
|  | **thiamine related** |  |
| *thiU* | thiamine-binding protein | 4.69 |
| *thiC* | phosphomethylpyrimidine synthase ThiC | 4.68 |
| *thiV* | thiamine permease | 3.90 |
| *tenA* | thiaminase II | 3.68 |
| *thiX* | thiamine permease | 3.66 |
| *thiW* | cobalt ABC transporter | 3.57 |
| *thiF* | thiamine biosynthesis protein ThiS | 3.35 |
| *thiD* | bifunctional hydroxymethylpyrimidine kinase/phosphomethylpyrimidine kinase | 3.31 |
| *thiO* | glycine oxidase | 3.20 |
| *thiG* | thiazole synthase | 3.10 |
| *tenI* | thiamine phosphate synthase | 3.05 |
| *thiS* | thiamine biosynthesis protein ThiS | 2.65 |
| *thiT* | thiamine transporter ThiT | 2.01 |
|  | **others** |  |
| *glpD* | aerobic glycerol-3-phosphate dehydrogenase | 2.45 |
| *ydbN* | hypothetical protein | 2.36 |
| *cidA* | holin-like protein CidA | 2.21 |
| *ssuB* | aliphatic sulfonate ABC transporter ATP-binding protein | 2.12 |
| Down regulated |  |  |
| *pstC* | ABC transporter permease | -2.79 |
| *nrgA* | ammonium transporter NrgA | -2.64 |
| *ytnP* | MBL fold metallo-hydrolase | -2.11 |
| *pucR* | purine catabolism regulatory protein | -2.03 |

*^a^*Differentially expressed was defined by a fold change of >2 or <-2 and an RPKM of >10.
