# Supplementary material for: Fungal mycelia and bacterial thiamine establish a mutualistic growth mechanism
Source: Life Sci Alliance. 2020 Sep 21;3(12):e202000878. doi: 10.26508/lsa.202000878 (PMC7574024; doi:10.26508/lsa.202000878)
Supplement: Supplementary file 20 [file LSA-2020-00878_TableS4.docx]

Table S4. strains used in this study

| Strain | Genotype | Source |
| --- | --- | --- |
| *A. nidulans* |  |  |
| TN02A3 | *pyrG89; argB2;* Δ*nkuA::argB; pyroA4* | (Nayak et al, 2006) |
| Δ*thiA* | *biA1*; *argB2*; Δ*thiA::argB* | (Shimizu et al, 2016) |
|  |  |  |
| *B. subtilis 3610* | Wild type | (Branda et al, 2001) |
| *B. subtilis 168* | *trpC2* | Lab stock |
| Δ*hag* | *168* Δ*hag::Cm^R^* | This study |
| Δ*thi-operon* | *168* Δ*thi-operon::Spec^R^* | This study |
| thi-rep | *3610* + pHY300MK  (Pveg-ZsGreen, PtenA-mScarlet-1:: *Tet^R^*) | This study |
