# Supplementary material for: Fungal mycelia and bacterial thiamine establish a mutualistic growth mechanism
Source: Life Sci Alliance. 2020 Sep 21;3(12):e202000878. doi: 10.26508/lsa.202000878 (PMC7574024; doi:10.26508/lsa.202000878)
Supplement: Supplementary file 21 [file LSA-2020-00878_TableS5.docx]

Table S5. Nucleotides used in this study

| Primer name | Sequence (5’ to 3’) |
| --- | --- |
| tenA-5 | GTAGGATCACCGGAGCATGAA |
| tenA-N5 | GCTTCGCGCACTGATTGAA |
| tenA-N3 | ctttatccaattttcGAAAATAAAAAAACCACTTTCCCGCAA |
| tenA-C5 | tcactaacctgccccGATCACCAGCTGATGGTGA |
| tenA-C3 | GGACGTGCCGCTTTGACAACA |
| tenA-3 | CACAAGCTCTCCGCCAAGGTA |
| Cm-Fw | GGTTTTTTTATTTTCgaaaattggataaagtgggatatttttaaaatatatattta |
| Cm-Rv | ATCACCATCAGCTGGTGATCggggcaggttagtgacatta |
| hag N5 | AGAGCCATTTGAAAAGTCTACTGC |
| hag-N3 | AGATCTCCATATAATTTTTGTGTTTTGTTCCTCCCTGAAT |
| spc-Fw | ATTCAGGGAGGAACAAAACACAAAAATTATATGGAGATCT |
| spc-Rv | CGCCAAGGTCTTTTTTAAAAAAGCTTCACTAAATTAAAGT |
| hag-C5 | ACTTTAATTTAGTGAAGCTTTTTTAAAAAAGACCTTGGCG |
| hag-C3 | AGACCTGTTATTCTTGTGACCATC |
| hag-3 | CTACAAATAACCCAAGAAATTCAG |
| tenA-5 | GTAGGATCACCGGAGCATGAA |
| tenA-mSca-Rv | TTCACCTTTAGATACcacaaatcattccccctctg |
